# Distinct cellular effects of myotonic dystrophy type 2 repeat-associated non-AUG tetrapeptides

**DOI:** 10.1242/dmm.052729

**Published:** 2026-05-18

**Authors:** Marta Marzullo, Assia De Simone, Marta Terribili, Michela Di Salvio, Degisew Yinur Mengistu, Maria Patrizia Somma, Rodrigo D'Amico, Gianluca Canettieri, Gianluca Cestra, Laura Ciapponi

**Affiliations:** ^1^ Department of Biology and Biotechnologies ‘C. Darwin’, Sapienza University of Rome, 00185 Rome, Italy; ^2^ Institute of Molecular Biology and Pathology, National Research Council (IBPM, CNR), c/o Department of Biology and Biotechnologies ‘C. Darwin’, Sapienza University of Rome, 00185 Rome, Italy; ^3^ Department of Molecular Medicine, Sapienza University of Rome, 00185 Rome, Italy

**Keywords:** *Drosophila melanogaster*, Myotonic dystrophy type 2, Repeat expansion disorders, Protein toxicity, QAGR, LPAC

## Abstract

Myotonic dystrophy type 2 (DM2) is an autosomal dominant, multisystemic disorder caused by the expansion of CCTG repeats in the first intron of the *CNBP* gene. Repeat-associated non-AUG (RAN) translation of the expanded CCTG RNA generates two tetrapeptide repeat proteins, polyQAGR and polyLPAC, but their roles in DM2 pathogenesis remain unclear. To define their individual contributions, we expressed codon-optimized polyQAGR and polyLPAC peptides with an ATG start codon in *Drosophila melanogaster*. Expression of both tetrapeptide repeat proteins reduced viability and lifespan and induced eye degeneration and locomotor defects. We found that polyQAGR accumulated in the nucleolus, disrupted nucleolar integrity and impaired rRNA processing. It also interfered with autophagy, promoting *Atg5* and *Atg7* transcription and accumulation of Atg8a- and Ref(2)P-positive aggregates. Overexpression of Atg8a or Ref(2)P mitigated polyQAGR-induced eye toxicity, whereas knockdown of autophagy genes worsened it. Conversely, PolyLPAC expression increased the cytoplasmic pool of TIAR in human cells and in DM2 patient-derived myoblasts. Together, these findings show that polyQAGR and polyLPAC exert distinct toxic effects that likely converge to drive DM2 pathogenesis and may represent promising therapeutic targets.

## Teaser

**Summary:** polyLPAC and QAGR repeat-associated non-AUG tetrapeptides cause distinct cellular toxicities in a *Drosophila* model of myotonic dystrophy type 2, driving disease via nucleolar stress, autophagy dysregulation and altered stress responses.

## INTRODUCTION

Myotonic dystrophy type 2 (DM2; OMIM 602668) is a multisystemic autosomal dominant disease that displays a wide spectrum of clinical manifestations, including proximal myotonia, degeneration of muscle fibres, defective cardiac conduction, cataracts, insulin resistance and other endocrine disorders (Meola, 2020; Meola and Cardani, 2015).

The genetic basis for DM2 is the expansion of an unstable CCTG repeat on chromosome 3q21, in the first intron of the cellular nucleic acid-binding protein (*CNBP*) gene, also named *ZNF9* (zinc finger protein 9; Liquori et al., 2001). The cause for the expansion is unknown; however, the expanded DM2 alleles are strongly unstable with significant increase in length over time. The size of the (CCTG)n expansion is <30 repeats in individuals without DM2, whereas in patients with DM2 it is >75 CCTG and can reach 11,000 repeats (Bachinski et al., 2009; Liquori et al., 2001).

Although the pathogenic mechanisms in DM2 are still not fully understood, several models have been proposed (Marzullo et al., 2023a). Accordingly, the CCTG expansion may affect CNBP expression *in cis* by altering transcription, impairing mRNA processing or causing nuclear sequestration of the transcripts. Haploinsufficiency of the *CNBP* gene has been suggested to play an important role in DM2 pathogenesis: indeed, mice carrying homozygous or heterozygous deletion of the *Cnbp* allele develop clinical manifestations strongly reminiscent of DM2 myopathy (Wei et al., 2018), and downregulation of CNBP expression in *Drosophila* muscle tissues causes severe locomotor defects that are rescued by reconstitution with either *Drosophila* or human CNBP (Coni et al., 2021). A gain-of-function RNA-mediated mechanism involves the transcription of CCTG repeats, resulting in the production of toxic CCUG-expanded RNA. This expanded RNA is associated with three primary pathological gain-of-function mechanisms: (1) formation of toxic RNA foci; (2) general splicing defects, related to sequestration and dysregulation of RNA-binding proteins, such as the muscleblind-like proteins (MBNL1-3), CUG-binding protein 1 (CUG-BP1; also known as CELF1) (Kanadia et al., 2006; Mohan et al., 2014) and the rbFOX proteins (Sellier et al., 2018); and (3) specific impaired CNBP pre-mRNA splicing, whereby the CCUG expansions potentially alter RNA structure and/or obstruct splicing regulators (Sznajder et al., 2018). Finally, repeat-associated non-AUG (RAN)-mediated translation has also been demonstrated (reviewed by Zu et al., 2018). Ectopic RAN translation has been reported in several degenerative diseases caused by microsatellite expansions, such as spinocerebellar ataxia type 8 (SCA8), fragile X-associated tremor ataxia syndrome (FXTAS), C9ORF72-mediated amyotrophic lateral sclerosis/frontotemporal dementia (ALS/FTD), Fuchs endothelial corneal dystrophy, SCA31, Huntington disease, DM1 and DM2 (reviewed by Guo et al., 2022).

In particular, the expanded DM2-CCTG repeats are transcribed bidirectionally, and the resulting RNAs undergo RAN translation, producing tetrapeptide repeat proteins (TPRs) of Pro-Ala-Cys-Leu (LPAC) sequence from the sense strand and Gln-Ala-Gly-Arg (QAGR) tetrapeptide from the antisense strand. Both repeated tetrapeptides accumulate in DM2 patient brain and skin tissues and seem to be responsible for at least some of the neurological symptoms of patients with DM2 (Rimoldi et al., 2025; Tusi et al., 2021; Zu et al., 2017).

Each of these potential mechanisms of toxicity is likely to contribute to disease initiation and progression; however, the extent to which each of them contributes to the development and clinical manifestations of the disease, and how they interact with each other, is unclear (Malik et al., 2021). It has recently been proposed that at early stages CNBP haploinsufficiency and toxic nuclear RNAs are the main pathogenic mechanisms, while later the mRNAs accumulate in the cytoplasm where RAN translation occurs leading to production of toxic tetrapeptides, which may contribute to the worsening of the phenotype (Malik et al., 2021; Marzullo et al., 2023a).

Interestingly, the accumulation of different RAN-translated peptides in distinct cellular compartments may lead to unique pathogenic mechanisms and, consequently, variable disease presentations and severities in microsatellite expansion disorders. Although the precise role of RAN-translated peptides remains under investigation, their discovery has added a new layer of complexity to these diseases, with evidence suggesting that they are toxic, aggregating proteins contributing to disease progression.

Despite these insights, the molecular mechanisms underlying the exact contribution of DM2 RAN peptides to cellular dysfunction and pathogenesis remains largely unknown. To address this, we characterized the effects of polyLPAC and QAGR tetrapeptides using *Drosophila melanogaster* as an *in vivo* model system. The two TPRs displayed distinct subcellular localizations and influenced separate cellular processes: QAGR accumulated in the nucleolus and modulated autophagy, whereas LPAC localized predominantly in the cytoplasm and promoted stress granule formation. These findings suggest that RAN-translated tetrapeptides contribute to DM2 pathology through discrete, peptide-specific cellular mechanisms.

## RESULTS

### Generation and characterization of DM2 TPRs transgenic flies

**Fig. 1.**
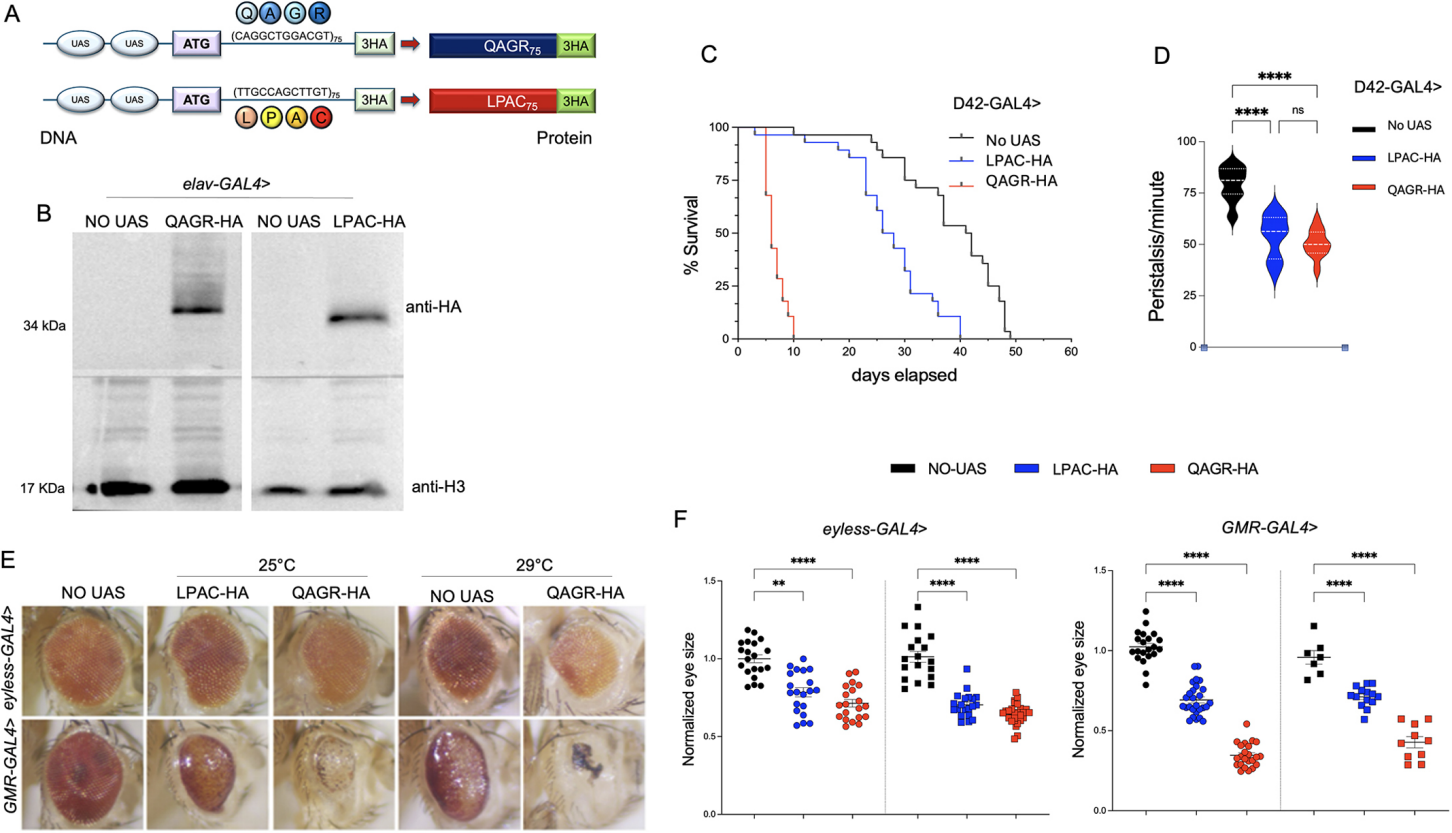
**Expression of DM2 tetrapeptide repeats in motor neurons reduces lifespan, impairs locomotion and causes eye degeneration.** (A) Schematic of transgenic ‘protein-only’ constructs that were generated using codons alternative to those found within the CCTG repeat arrays. Expansions were led by a canonical ATG codons, tagged with a triple HA epitope (3HA) and expressed under the control of the GAL4/UAS system. (B) Immunoblot of protein extracts obtained from *elav*-GAL4> UAS-LPAC (LPAC-HA) or UAS-QAGR (QAGR-HA) larval brains, labelled with anti-HA antibody to detect the transgenic proteins and anti-H3 as loading control. The progeny of wild-type individuals crossed to the driver (NO UAS) were used as control. (C) Survival rate as a function of time for flies expressing DM2 repeats under the control of the *D42*-GAL4 driver, showing a drastic reduction in lifespan of flies expressing polyQAGR (red), and a milder yet noteworthy effect upon polyLPAC expression (blue), compared to controls (NO UAS, black); *n*≥30 animals for each genotype. (D) Graphical representation of the distribution of peristaltic contractions performed in 1 min by third instar larvae expressing UAS-LPAC (blue), UAS-QAGR (red) or NO UAS (black) under the control of *D42*-GAL4 driver at 25°C. *n*≥15 larvae tested for each genotype in at least three independent experiments. (E) Representative adult eyes of flies expressing either UAS-LPAC or UAS-QAGR under the control of the *GMR*- or *eyeless*-GAL4 driver. Flies were mated and reared at 25°C or 29°C, as indicated. (F) Statistical analysis of eye area of the same genotypes shown in E. The *Drosophila* eye arbitrary size was measured using Fiji. Each dot represents a single eye, *n*≥20. As controls, progeny of wild-type individuals crossed to the driver (NO UAS) were used. ****P<0.0001, **P<0.001 (one-way ANOVA). ns, not significant. Error bars represent s.e.m.

To analyse the specific contribution of the DM2 TPRs *in vivo*, we generated transgenic flies bearing a codon-optimized sequence that differs from the natural CCTG sequence and that encodes either the LPAC (TTGCCAGCTTGT) or the QAGR (CAGGCTGGACGT) protein repeats (75 repetitions each; Fig. 1A). The coding sequences were placed under the control of an upstream activating sequence (UAS)-inducible element, after a canonical ATG starting codon, and C-terminal tagged with a 3HA peptide (Fig. 1A). Therefore, different GAL4 drivers were used to induce the expression of 75 repeats of LPAC or QAGR HA-tagged tetrapeptides in different tissues.

To assess the expression efficacy of the transgenes, we performed immunoblotting analysis on protein extracts from larval brains expressing either UAS-(QAGR)_75_-3HA or UAS-(LPAC)_75_-3HA (hereafter referred to as UAS-QAGR and UAS-LPAC, respectively) under the pan-neuronal driver *elav*-GAL4. The results obtained showed that both TPRs are expressed at significant levels (Fig. 1B). Therefore, this transgenic fly model is well-suited to investigate the specific cellular and molecular mechanisms of TPR-based toxicity and its role in DM2 pathogenesis.

### DM2 TPRs reduce lifespan, impair locomotion and induce eye degeneration

To address the toxic effect of DM2 tetrapeptides in cells involved in the control of muscle contraction, we analysed the phenotypic consequences of polyLPAC and polyQAGR expression specifically within motor neurons, using the *D42*-GAL4 driver. Flies expressing QAGR TPRs in motor neurons exhibited a significant reduction in their lifespan with a median survival time of 6 days for QAGR compared to 41.5 days for wild-type controls. The impact of LPAC repeats was less pronounced, but still significant compared to controls, with a median lifespan of 27 days (Fig. 1C). Interestingly, TPR toxicity also affected larval locomotor capabilities. Larval locomotion is characterized by peristaltic waves of muscle contractions that propagate along the body through coordinated segmental movements. Expression of either polyLPAC or polyQAGR in motor neurons significantly reduced peristaltic movements compared to controls (Fig. 1D).

The *Drosophila* eye provides an ideal tissue for studying the genetic regulation of neurodegeneration and the effect of the expression of toxic products. Moreover, eye expression of pure, uninterrupted CCUG-repeat expansions has been shown to cause strong retina degeneration (Mishra and Knust, 2013; Yu et al., 2015). Thus, we investigated whether the expression of LPAC and QAGR DM2 TPRs early (*ey*-GAL4) or late (*GMR*-GAL4) during eye development causes retina degeneration. Expression of either the UAS-LPAC or the UAS-QAGR construct under the control of eye-specific drivers led to a significant reduction in eye area, abnormal pigmentation, and a smooth eye surface, indicative of neurodegeneration and strongly resembling the phenotypes observed in previous fly models of DM2 (Fig. 1E,F) (Yenigun et al., 2017; Yu et al., 2015).

### Subcellular localization of LPAC and QAGR TPRs

**Fig. 2.**
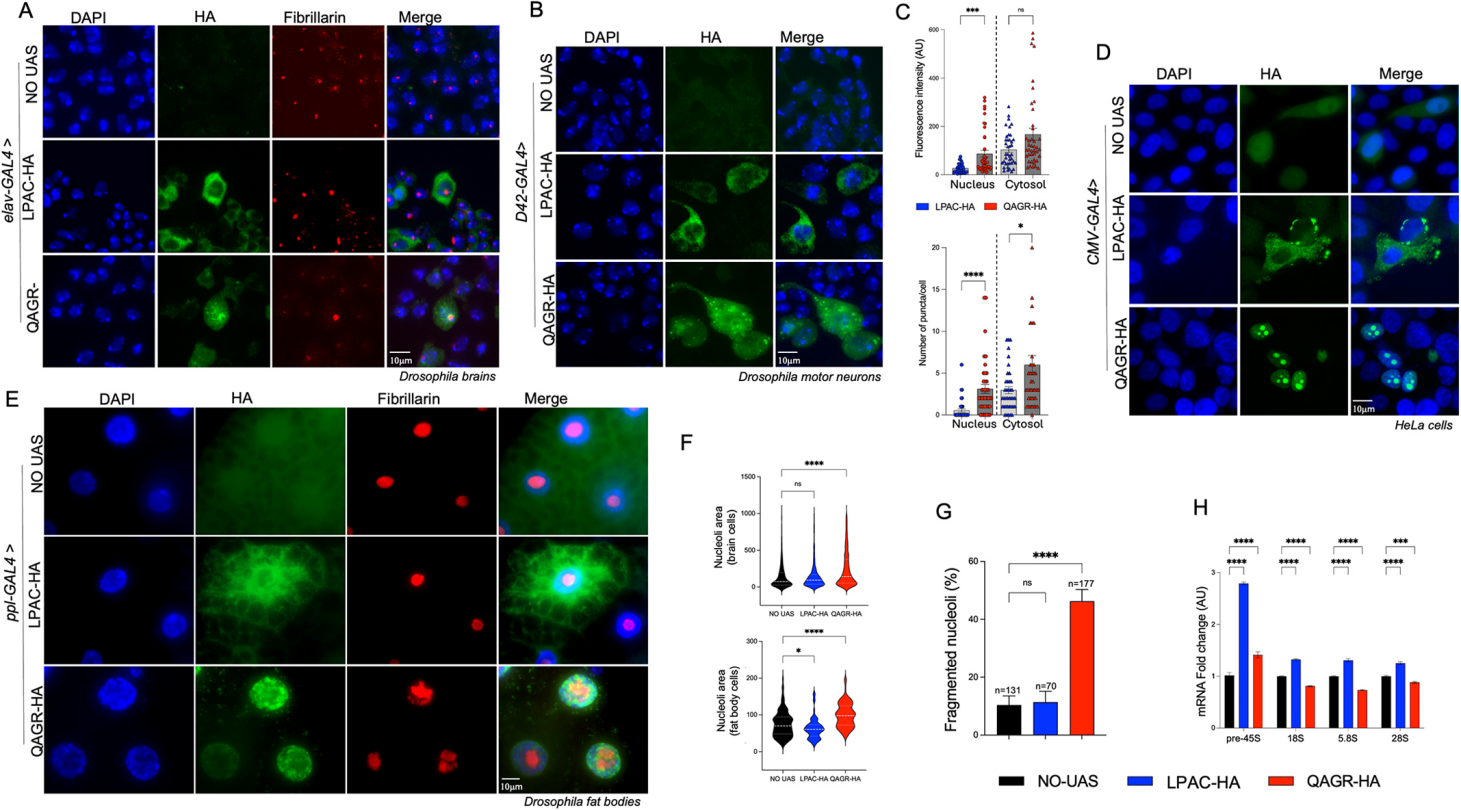
**QAGR TPRs expression induces nucleolar stress.** (A) Representative images of *Drosophila* larval brain cells from controls (NO UAS) or from larvae expressing either the UAS-LPAC (LPAC-HA) or the UAS-QAGR (QAGR-HA) construct, under the control of the *elav*-GAL4 driver. Cells were stained with DAPI (blue), anti-HA (green) and anti-fibrillarin (red) antibodies. Note the distinct cellular expression patterns between the two tetrapeptides, with polyLPAC leading to a primarily cytoplasmic diffuse localization and polyQAGR forming aggregates around the nucleolus. (B) Representative images of *Drosophila* larval motor neurons from controls (NO UAS) or from larvae expressing either UAS-LPAC or UAS-QAGR tetrapeptide repeats, under the control of *D42*-GAL4. Cells were stained with DAPI (blue) and anti-HA antibody (red). As controls, progeny of wild-type individuals crossed to the driver (NO UAS) were used. (C) Quantification of fluorescence intensity signal (top) or number of aggregates per cell (bottom) for polyLPAC (blue symbols) and polyQAGR (red symbols) in the nucleus (circles) or in the cytoplasm (triangles), as shown in A. Each dot represents a single cell (*n*>40 for fluorescence intensity; *n*>38 for aggregate counts). (D) Representative images of HeLa cells transfected with either the UAS-LPAC or the UAS-QAGR plasmid together with the *pCMV*-GAL4 plasmid, stained with DAPI (blue) and anti-HA antibody (green). Cell transfected only with *pCMV*-GAL4 plasmid were used as control (NO UAS). Note that polyLPAC exclusively localizes to the cytoplasm, whereas polyQAGR is specifically distributed in the nucleolus. (E) Representative larval fat bodies from individual animals expressing either the UAS-LPAC or the UAS-QAGR construct, under the control of the *ppl*-GAL4 driver stained with anti-HA (green) and fibrillarin (red) antibodies, and DAPI (blue). (F) Quantification of nucleolar size in larval brain cells (top; *n*>800) and in fat body cells (bottom; *n*>75), as in A and E, respectively. In fat bodies, nucleolar size measurements were restricted to structures with an area ≥15 µm², to exclude fragmented nucleolar particles while preserving physiological size variability (cut off 15 µm²). (G) Quantification of the percentage of fragmented nucleoli in fat body cells as in E; *n* represents the total number of nuclei scored. (H) Quantitative RT-PCR on total RNA extracts from UAS-LPAC- or UAS-QAGR-expressing larvae under the control of the *ppl*-GAL4 driver. *n*=2 biological replicates and *n*=6 technical replicates. As controls, progeny of wild-type individuals crossed to the driver (NO UAS) were used. ****P<0.0001, ***P<0.001, *P<0.01 (in F-H, one-way ANOVA; in C, Mann–Whitney test for two-group comparisons: nuclear polyLPAC versus nuclear polyQAGR and cytoplasmic polyLPAC versus cytoplasmic polyQAGR). ns, not significant. Error bars represent s.e.m. AU, arbitrary units. Scale bars: 10 µm.

Immunofluorescence analysis on larval brains cells expressing the transgenes under the pan-neuronal driver *elav*-GAL4 showed that polyLPAC was predominantly cytoplasmic, exhibiting both diffuse and punctate staining. Conversely, polyQAGR displayed a distinct nuclear pattern, with specific staining around the nucleolus, as indicated by its proximity with the well-established nucleolar marker fibrillarin (Fig. 2A). Similar observations were obtained in brain cells from larvae expressing the transgenes under the motor neuronal driver *D42*-GAL4 (Fig. 2B,C).

Quantitative analysis revealed distinct subcellular localization of the two TPRs, with polyLPAC predominantly confined to the cytoplasm, whereas polyQAGR was distributed between the cytoplasm and nucleus. Consistent with this distribution, we showed that QAGR repeats formed a significantly higher number of aggregates compared to LPAC TPRs in both the nucleus and cytoplasm, with a more pronounced difference in the nucleus (Fig. 2B,C). As the No-UAS control does not express HA, all measurements were based on a direct comparison between the two TPRs, thereby emphasizing their divergent localization profiles. Analogous distinct subcellular localization patterns were observed in HeLa cells (Fig. 2D), in agreement with previous observations in transfected HEK293T cells (Zu et al., 2017).

### Expression of QAGR TPRs induces nucleolar stress and affects autophagy

In *Drosophila* fat bodies, nucleolar defects can have systemic effects, including an indirect impact on muscle function, likely due to altered protein synthesis, energy metabolism and stress signalling. Further, as fat body cells are highly engaged in metabolic and immune functions, their nucleoli are typically well-developed to support high ribosome production. Immunofluorescence analysis of fat body cells expressing UAS-QAGR under the control of the specific driver *ppl*-GAL4 confirmed that QAGR TPRs localize both in the nucleus and in cytoplasm, in line with the phenotype observed in larval brains. Specifically, we confirmed the peri-nucleolar localization of polyQAGR as ascertained by co-labelling with the nucleolar marker fibrillarin (Fig. 2E). Furthermore, expression of polyQAGR led to altered nucleolar morphology, evident nucleolar fragmentation and significantly increased nucleolar size (Fig. 2E-G).

Since perturbation of nucleolar morphology could potentially impair ribosomal RNA biogenesis, we measured the levels of 5S, 18S and 28S rRNAs, which are processed from the precursor 45S rRNA. qRT-PCR analysis revealed a significant decrease in the levels of mature 5.8S, 18S and 28S rRNAs in larvae expressing polyQAGR, accompanied by a marked accumulation of pre-rRNA 45S compared to controls (Fig. 2H).

These results suggest that expression of polyQAGR affect pre-rRNA processing and maturation, impairing nucleolar function. In contrast, immunofluorescence analysis of fat body cells expressing UAS-LPAC under the control of *ppl*-GAL4 confirmed the cytoplasmatic localization observed in larval brains, but showed that LPAC TPRs had no detectable effect on nucleolar morphology, although nucleoli were slightly smaller than controls (but not in larval brain cells; Fig. 2F,G). Notably polyLPAC expression caused a general upregulation of both pre-rRNA 45S and mature rRNAs (Fig. 2H), suggesting that polyLPAC induces a stress response that enhances rDNA transcription and rRNA processing without perturbing nucleolar integrity.

To assess the effects of the DM2 TPRs in muscle, the most affected tissue in patients with DM2, we expressed LPAC or QAGR tetrapeptide repeats under the control of the muscle-specific *Mef2-*GAL4 driver. Notably, expression of both TPRs in muscle led to adult lethality at 25°C. Lowering the temperature to 18°C enabled polyQAGR-expressing third instar larvae to be obtained, whereas polyLPAC expression remained lethal even under these conditions.

**Fig. 3.**
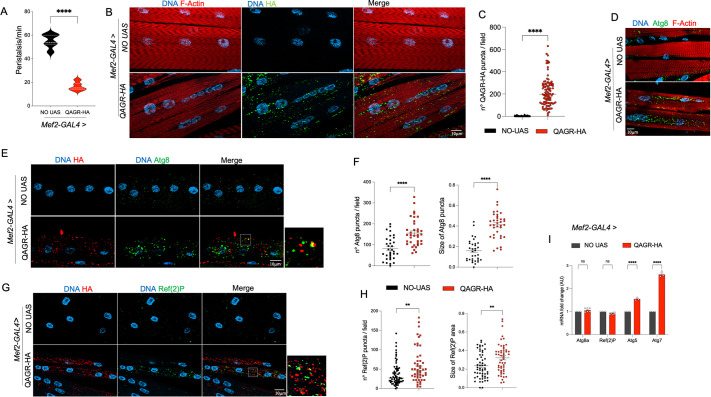
**QAGR-TPRs form aggregates in larval muscles, affecting locomotor activity and autophagy.** (A) Graphical representation of the number of peristaltic contractions performed in 1 min by control larvae (NO UAS; black) or UAS-QAGR-expressing larvae (red). *n*≥10 larvae tested for each genotype. The low number of larvae used for the experiment is justified by the semi-lethality of the informative progeny. (B) Representative confocal images of third instar larval muscle fibres expressing or not (NO UAS) UAS-QAGR under the control of the muscle-specific driver *Mef2-*GAL4 at 18°C stained with anti-HA (green) and anti-phalloidin (a marker for F-actin; red) antibodies, and DAPI (DNA; blue). (C) Graphical representation of the number of QAGR puncta/field (rounds) present in control larval muscles (NO UAS; black) and in UAS-QAGR-expressing larval muscle (red). *n*≥100 fields tested for each genotype. As controls, progeny of wild-type individuals crossed to the driver (NO UAS) were used. (D) Representative confocal images of third instar larval muscle fibres expressing, or not (NO UAS), UAS-QAGR under the control of the muscle-specific driver *Mef2-*GAL4 at 18°C. Larval muscles were stained with fluorescence-labelled phalloidin, a marker for F-actin (red), anti-Atg8a antibody (in green) and DAPI (in blue). Note that the polyQAGR-expressing muscle fibres display a high number of Atg8a-positive puncta. (E) Representative confocal images of third instar larval muscle fibres expressing, or not (NO UAS), UAS-QAGR under the control of the muscle-specific driver *Mef2-*GAL4 at 18°C. Larval muscles were stained with anti-HA (red) and anti-Atg8a (green) antibodies and DAPI (DNA; blue). Dashed square represents the area shown at high magnification on the right. (F) Graphical representation of the number (squares) and size (circles; in µm) of Atg8a puncta present in control larval muscles (NO UAS; black) and in UAS-QAGR-expressing larval muscle (red). *n*≥30 fields tested for each genotype. (G) Representative confocal images of third instar larval muscle fibres expressing, or not (NO UAS), UAS-QAGR under the control of the muscle-specific driver *Mef2-*GAL4 at 18°C. Larval muscles were stained with anti-HA (red) and anti-Ref(2)P (green) antibodies, and DAPI (DNA; blue). (H) Graphical representation of the number (squares) and size (circles) of Ref(2)P puncta present in UAS-QAGR-expressing larval muscle (red) compared to controls (NO UAS, black). *n*≥50 fields tested for each genotype. (I) qRT-PCR analysis of the *Atg8*, *Atg5*, *Atg7* and *ref(2)P* genes on RNAs obtained from control larvae (NO UAS) or from larvae expressing UAS-QAGR under the control of the *Mef2*-GAL4 driver. As controls, progeny of wild-type individuals crossed to the driver (NO UAS) were used. ****P<0.0001, **P<0.001 (in A,C,F,H, unpaired one-tailed *t*-test; in I, two-way ANOVA). ns, not significant. Error bars represent s.e.m. AU, arbitrary units. Scale bars: 10 µm.

As shown in Fig. 3A, larvae expressing polyQAGR in muscles exhibited severe locomotor defects, evidenced by a significant reduction in peristaltic wave frequency. These defects were associated with the accumulation of QAGR TPR aggregates in muscle tissue, as revealed by multiple HA-positive inclusions detected by immunofluorescence in larval muscle fillets (Fig. 3B,C).

Because the accumulation of toxic peptides is often associated with autophagy, to elucidate how polyQAGR aggregation contributes to larval locomotor defects we investigated whether these peptides affect autophagy, a process that plays a crucial role in the clearance of toxic protein aggregates and damaged organelles (Boivin et al., 2020; Ciechanover and Kwon, 2015).

The microtubule-associated protein light chain 3 variants (LC3s) and their paralogues, GABARAPs, are cleaved, processed, and inserted into nascent autophagosomes, where they participate in autophagosome formation and cargo selection for degradation (Lamark and Johansen, 2021). In *Drosophila* the Autophagy-related gene 8a (Atg8a) protein serves as the functional homologue of both human LC3 and GABARAP and is widely used as a marker of autophagic vesicles (Jipa et al., 2021).

**Fig. 4.**
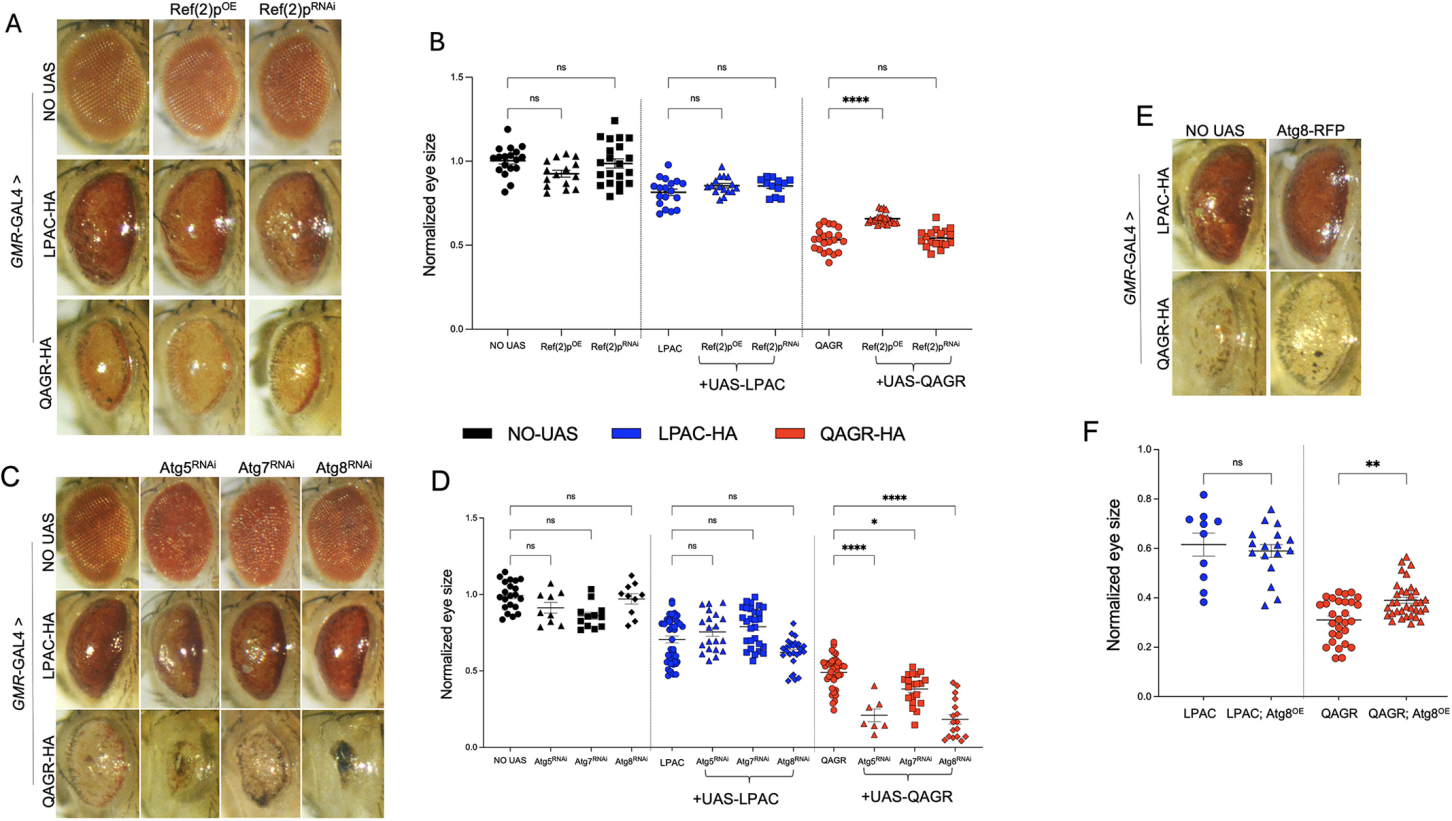
**polyQAGR-induced eye degeneration is influenced by autophagic gene modulation.** (A) Representative adult eyes of flies expressing, or not (NO UAS), either UAS-LPAC or UAS-QAGR under the control of the *GMR*-GAL4 driver, alone or in combination with either Ref(2)P silencing [UAS-Ref(2)P^RNAi^] or overexpression [UAS-Ref(2)P^OE^]. Flies were mated and reared at 25°C. (B) Graphical representation of the mean eye size of the same genotypes shown in A. *Drosophila* eye size was measured using Fiji. *n*≥15. (C) Representative adult eyes of flies expressing, or not (NO UAS), either UAS-LPAC or UAS-QAGR under the control of the *GMR*-GAL4 driver, alone or in combination with either Atg5 (UAS-Atg5^RNAi^), Atg7 (UAS-Atg7^RNAi^) or Atg8a (UAS-Atg8^RNAi^) silencing. Flies were mated and reared at 25°C. (D) Graphical representation of the mean eye size of the same genotypes shown in A. *n*≥20. (E) Representative adult eyes of flies expressing, or not (NO UAS), either UAS-LPAC or UAS-QAGR under the control of the *GMR*-GAL4 driver, alone or in combination with Atg8 overexpression (UAS-Atg8^OE^). Flies were mated and reared at 25°C. (F) Graphical representation of the mean eye size of the same genotypes shown in E. *n*≥20. *Drosophila* eye size was measured using Fiji. As controls, progeny of wild-type individuals crossed to the driver (NO UAS) were used. ****P<0.0001, **P<0.001, *P<0.01 (one-way ANOVA). ns, not significant. Error bars represent s.e.m.

To study autophagosome activity upon TPR expression, we performed immunostaining of larval muscles expressing polyQAGR using an antibody that labels fly Atg8a. This analysis revealed a marked increase in the number of Atg8a-immunoreactive puncta in larval muscles expressing UAS-QAGR under the *Mef2*-GAL4 control, compared to controls (Fig. 3D-F). This phenotype closely resembles that observed under starvation conditions, suggesting that polyQAGR expression may activate the autophagy pathway, mimicking a nutrient-deprivation phenotype (Allen et al., 2020; Érdi et al., 2012; Scott et al., 2004; Zirin et al., 2015). Immunofluorescence staining for Ref(2)P, the fly homologue of the selective autophagy receptor p62 (Nezis et al., 2008), revealed also a significant increase in Ref(2)P-positive puncta in QAGR-expressing muscles compared with controls (Fig. 3G,H). Notably, both Atg8a- and Ref(2)P-positive puncta were not only more numerous but also increased in size following QAGR expression (Fig. 3E-H). However, RT-qPCR experiments revealed no significant changes in *Atg8a* or *ref*(*2)P* transcript levels upon polyQAGR expression, indicating that their accumulation is not driven by their transcriptional upregulation (Fig. 3I). Together, these findings indicate that polyQAGR expression challenges autophagy, causing both accumulation and enlargement of autophagic vesicles, without detectable changes at the transcriptional level. In contrast, transcription of the key regulators of autophagy *Atg5* and *Atg7* (Mizushima et al., 2011) was significantly upregulated, highlighting a selective transcriptional response within the autophagy pathway (Fig. 3I). Consistent with these results, RNAi-mediated downregulation of Atg8 exacerbated polyQAGR-mediated toxicity (Fig. 4A-D), while overexpression of Atg8a or Ref(2)P significantly ameliorated eye degeneration (Figs 4A,B,E,F). In line with this, knockdown of *Atg5* or *Atg7* significantly worsened polyQAGR-induced eye degeneration (Fig. 4C,D). Together, these data suggest that polyQAGR expression elicits an autophagy-related response and that genetic enhancement of autophagy components mitigates polyQAGR-mediated toxicity.

### LPAC TPRs associate with stress granules

**Fig. 5.**
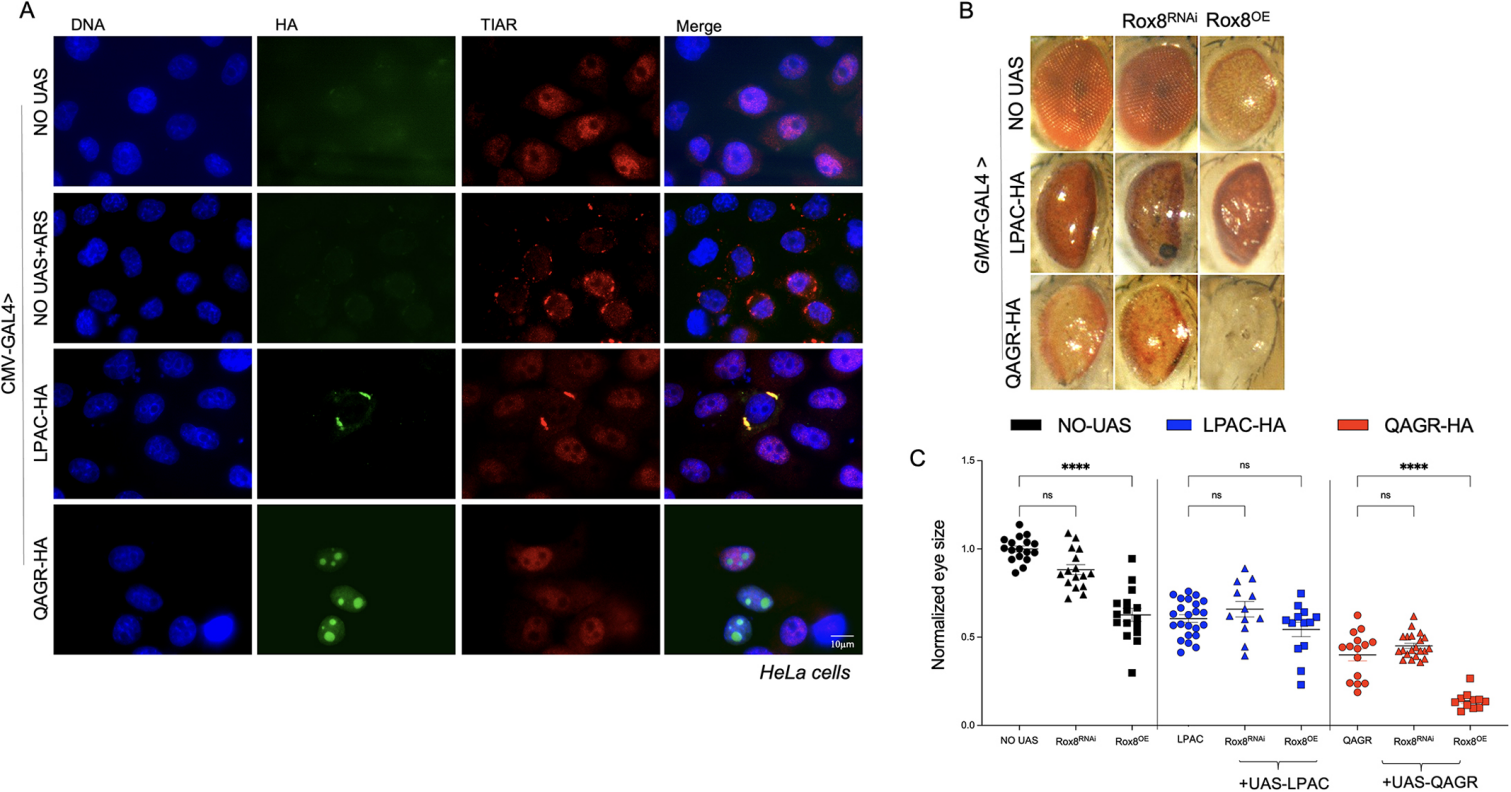
**LPAC TPRs colocalize with TIAR in HeLa cells.** (A) Representative images of HeLa cells transfected with either UAS-LPAC or UAS-QAGR plasmid together with the *pCMV*-GAL4 plasmid stained with anti-HA (green) and anti-TIAR antibodies (red), and DAPI (DNA; blue). HeLa cells transfected only with the *pCMV*-GAL4 plasmid treated or not with arsenite, were used as positive and negative control, respectively. Scale bar: 10 µm. (B) Representative adult eyes of flies expressing, or not (NO UAS), either UAS-LPAC or UAS-QAGR under the control of the *GMR*-GAL4 driver, alone or in combination with either UAS-Rox8^RNAi^ or UAS-Rox8^OE^. Flies were mated and reared at 25°C. (C) Graphical representation of the mean eye size of the same genotypes shown in B. *n*≥20. *Drosophila* eye size was measured using Fiji. As controls, progeny of wild-type individuals crossed to the driver (NO UAS) were used. ****P<0.0001 (one-way ANOVA). ns, not significant. Error bars represent s.e.m.

Our observations on fly eye neurodegeneration suggest that polyLPAC-mediated toxicity is not affected by modulation of the autophagy factors (Fig. 4). Interestingly, LPAC TPRs primarily localized in the cytoplasm, where they occasionally formed granules (Fig. 2). Dipeptide repeat proteins (DPRs) such as polyGA, polyGP and polyPA, generated by RAN translation from the expanded CCCGGG repeat in the *C9orf72* gene, have been shown to induce stress granule (SG) formation (Tao et al., 2015). Thus, we assessed whether LPAC TPRs expression contribute to SG formation. To determine whether polyLPAC-containing granules colocalized with SGs, we performed co-immunofluorescence with antibodies against TIAR (TIAL2), an RNA-binding protein essential for SG assembly. To promote SG formation, we treated HeLa cells with sodium arsenite, which induces prominent oxidative stress that activates eIF2α phosphorylation, leading to global translational arrest (Jain et al., 2016). In untreated cells, TIAR typically localizes in the nucleus, whereas after sodium arsenite treatment it translocates to the cytoplasm where it promotes SG formation. Interestingly, in cells expressing polyLPAC, TIAR-positive cytoplasmic SGs colocalized with LPAC signals even in the absence of arsenite treatment (Fig. 5A). This suggests a link between LPAC repeats expression and SG formation, which may contribute to DM2 pathogenesis. Conversely, no SG activation was observed upon polyQAGR peptide expression (Fig. 5A).

Genetic interaction experiments with Rox8, the *Drosophila* orthologue of human TIAR, showed that modulation of Rox8 levels (either by silencing or overexpression) did not significantly modify the polyLPAC-induced eye degeneration. These findings suggest that, under our experimental conditions, changes in TIAR/Rox8 levels do not influence the severity of polyLPAC-associated toxicity. In contrast, Rox8 overexpression, which causes eye neurodegeneration, exacerbated the eye degeneration induced by polyQAGR, suggesting that perturbations of SG dynamics may have additive effects on the autophagy-related toxicity of QAGR TPRs (Fig. 5B,C).

### LPAC TPRs accumulate in the cytoplasm of human DM2 myoblasts

**Fig. 6.**
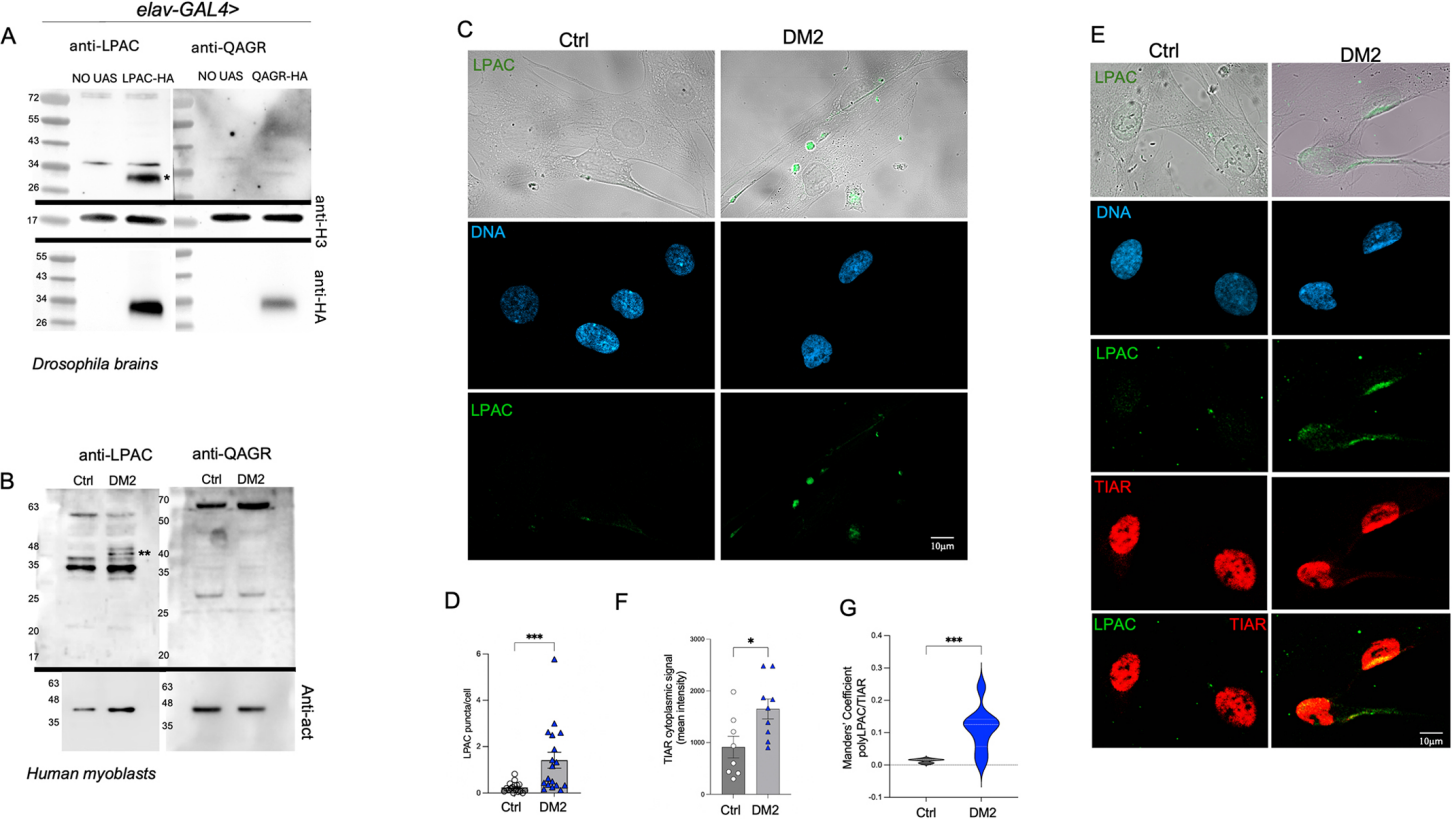
**LPAC TPRs accumulate in the cytoplasm of human DM2 myoblasts.** (A) Immunoblot of *Drosophila* protein extracts from transgenic larval brains expressing either UAS-LPAC or UAS-QAGR TPRs constructs under the control of the *elav*-GAL4 driver, labelled with anti-polyQAGR or anti-polyLPAC antibodies. H3 was used as loading control. The asterisk indicates the specific polyLPAC band detected in extracts from brains UAS-LPAC transgenic larvae (expected molecular weight ∼35 kDa). As controls, progeny of wild-type individuals crossed to the driver (NO UAS) were used. (B) Immunoblot of myoblast protein extracts from control and DM2 patients, labelled with anti-polyQAGR or anti-polyLPAC antibodies. Actin was used as loading control. Double asterisks indicate the specific polyLPAC band detected in protein extracts from DM2 patient-derived cells. (C) Representative images of human myoblasts from a single control individual and a single patient with DM2, stained with Hoechst (blue) and anti-polyLPAC antibody (green). (D) Graphical representation of the number of polyLPAC-positive puncta observed in control (white circles) or DM2 (blue circles) myoblasts, as shown in C. Each dot represents one field containing at least four cells. *n*≥100. (E) Representative images of human myoblasts from a control individual and a single patient with DM2 stained with Hoechst (blue), anti-polyLPAC (green) and anti-TIAR (red). (F) Graphical representation of TIAR cytoplasmic intensity quantified in control (white circles) and DM2 human myoblasts (blue circles), as shown in E. Each dot represents one field containing at least four cells. *n*≥80. (G) Violin plot of the Manders correlation coefficient between polyLPAC and TIAR signals observed in control (dark grey) and DM2 patient-derived myoblasts (blue). ***P<0.001, *P<0.05 (by Mann–Whitney test). ns, not significant. Error bars represent s.e.m. All myoblast experiments were performed using cells derived from a single control individual and a single patient with DM2; therefore, statistical analysis reflects technical replicates from independent imaging fields and culture batches rather than biological replication across individuals. Scale bars: 10 µm.

To determine whether DM2 RAN proteins are expressed *in vivo* in muscle cells of patients with DM2, we used commercial polyclonal antibodies targeting the DM2 LPAC and QAGR repeat motifs (Rösing et al., 2024). Immunoblot analysis showed that, in *Drosophila* protein extracts, although the anti-polyLPAC antibody recognized transgenic 3HA-tagged proteins, the anti-polyQAGR antibody did not label them (Fig. 6A). A similar pattern was observed in protein extracts from human myoblasts obtained from a single patient with DM2 or a single individual without DM2, where specific bands were detected for polyLPAC peptides in DM2 patient-derived cells, but not for polyQAGR (Fig. 6B), consistent with previous findings (Rösing et al., 2024).

The DM2-RAN proteins were previously shown to accumulate in brains and skin biopsies of patients with DM2 (Rösing et al., 2024; Zu et al., 2017); thus, we tested DM2 protein expression and localization in DM2 patient myoblasts. Immunofluorescence analysis further confirmed the absence of a detectable polyQAGR signal in DM2 patient myoblasts, whereas staining with the anti-polyLPAC antibody revealed the formation of specific cytoplasmic aggregates, significantly higher in DM2 cells in both number and size with respect to control myoblasts (Fig. 6C,D). We also performed double immunofluorescence analyses using antibodies against polyLPAC and the SG marker TIAR in both control and DM2-derived human myoblasts. In DM2 cells, TIAR showed a more cytoplasmic localization compared to control cells (Fig. 6F). Moreover, polyLPAC partially colocalized with cytoplasmic TIAR-positive staining (Fig. 6E,G), suggesting that polyLPAC RAN translation in DM2 patient-derived cells may be associated with a cellular stress response.

## DISCUSSION

The use of a codon-optimized *Drosophila* model expressing LPAC and QAGR tetrapeptide repeats offers an incisive tool to dissect the protein-specific toxicity of DM2 RAN translation products. Crucially, the ‘protein-only’ strategy circumvents sequence-specific RNA-mediated effects, enabling a direct analysis of pathological mechanisms driven by repeat-encoded peptides. Thus, our study provides significant insights into the contribution of DM2 RAN-translated TPRs to disease pathology.

A major finding is the distinct subcellular localization and aggregation tendencies of LPAC and QAGR TPRs. While polyLPAC primarily exhibited a cytoplasmic distribution with occasional aggregates, polyQAGR was present in both the nucleus and the cytoplasm where it frequently formed aggregates. This may be due to the high density of repetitive arginine residues, which enhance cell permeability and nuclear import by mimicking the positive charge typical of nuclear localization signals (Kwon et al., 2014). The nuclear localization of QAGR TPRs, particularly their close proximity with the nucleolus, suggests an effect on the nucleolar function. This is further supported by the alterations observed in the nucleolar morphology, as well as a reduction in 18S and 28S rRNA levels upon polyQAGR expression.

The extensive work on the arginine-rich polyGR and polyPR peptides in C9orf72 ALS/FTD provides a strong mechanistic parallel to our study of polyQAGR in DM2. The high density of arginine residues shared between polyQAGR and polyGR/polyPR suggest common pathogenic pathways, including enhanced nuclear import, nucleolar accumulation, and alteration of ribosomal RNA biogenesis. Indeed, polyPR has been shown to disturb normal nuclear protein organization and to promote aggregation, contributing to cellular stress and dysfunction, illustrating how toxic peptides can interfere with nuclear processes and protein homeostasis (Agnihotri et al., 2025). Thus, the parallel between polyQAGR and polyGR/polyPR highlights the importance of further investigating nucleolar targets and RNA-processing pathways affected by polyQAGR, potentially providing new insights into the role of nucleolar stress as a relevant mechanism of toxicity in DM2 pathophysiology (Hartmann et al., 2018; Kwon et al., 2014; Tao et al., 2015).

Conversely, the expression of LPAC TPRs causes a significant upregulation of unprocessed pre-45S rRNA. Since nucleolar accumulation of unprocessed pre-45S rRNA is a well-known cellular stress response associated with inhibition of global mRNA translation (Szaflarski et al., 2022), this polyLPAC-mediated effect on ribosomal biogenesis is in agreement with its possible role on SG formation. Accordingly, LPAC TPRs colocalize with SG components under basal conditions. The ability of polyLPAC to induce SG formation even in the absence of oxidative stress indicates an intrinsic capacity to alter RNA metabolism and translational regulation, potentially via aberrant recruitment of RNA-binding proteins such as TIAR. Although exogenous expression of polyLPAC could potentially induce massive stress responses due to high expression levels, which may not fully reflect the situation in DM2 patient-derived cells, we observed partial colocalization of polyLPAC signals with the cytoplasmic pool of TIAR in DM2 patient-derived cells. This finding supports our results obtained in flies and raises the possibility that RAN translation of polyLPAC in DM2 cells is associated with a cellular stress response.

A further important aspect of our study is the tissue-specific impact of polyQAGR expression in muscle, where it leads to clear dysregulation of the autophagy pathway. PolyQAGR expression is associated with the accumulation of Atg8a and Ref(2)P proteins without corresponding changes in their transcript levels, despite transcriptional upregulation of other autophagy factors, such as *Atg5* and *Atg7*. This suggests that, although autophagy is transcriptionally activated, it may be functionally compromised at the level of cargo processing or turnover.

In line with this interpretation, genetic reduction of *Atg5* or *Atg7* significantly worsens polyQAGR-induced eye degeneration. By contrast, *Atg8* or *ref(2)P* overexpression partially rescues the phenotype, supporting the idea that Atg8- and Ref(2)P-positive structures retain a protective function in the clearance of toxic species. Taken together, these findings support a working model in which polyQAGR expression elicits an autophagy response in muscle that becomes overwhelmed or functionally insufficient, resulting in inefficient clearance of toxic protein species. Partial rescue upon enhancement of specific autophagy components suggests that increasing autophagic capacity can buffer proteotoxic stress.

Accumulation of repeated peptide with consequent activation of autophagy has been reported in several diseases caused by microsatellite expansions such as FXTAS, ALS/FTD and Huntington disease (Alvarez-Mora et al., 2023; Ciechanover and Kwon, 2015; Cui et al., 2024; Mengistu et al., 2025; Pircs et al., 2018).

Upregulating autophagy aims to promote the clearance of toxic protein aggregates that contribute to the loss of neuron or muscle homeostasis. Although autophagy regulation is complex, its activation helps maintain cellular balance disrupted by pathogenic proteins. The selective modulation of specific steps of the autophagy process, such as autophagosome formation, cargo recognition, or fusion with lysosomes, can represent a promising strategy to reduce aggregate-mediated toxicity and slow disease progression.

In contrast to polyQAGR, polyLPAC-mediated toxicity is not affected by modulation of autophagy genes. Instead, it influences SG dynamics, which may still be relevant in contexts of cellular stress or ageing, when SG misregulation contributes to disease pathogenesis.

The association of LPAC peptides with SGs is particularly intriguing, as SG formation is a common cellular response to stress and has been implicated in several neurodegenerative diseases (Cui et al., 2024).

The analysis of the human DM2 patient-derived myoblast line further supports the relevance of our *Drosophila* model, as polyLPAC peptides formed cytoplasmic aggregates and colocalized with TIAR. Unfortunately, polyQAGR peptides were undetectable, because of antibody inefficacy.

In conclusion, our study highlights the distinct toxic contributions of LPAC and QAGR TPRs to DM2 pathology. The differential effects of these peptides on nucleolar function, autophagy, and SG dynamics highlight the complexity of DM2 pathogenesis and offer potential avenues for therapeutic intervention. Future studies should focus on elucidating the precise molecular mechanisms underlying these toxic effects and exploring targeted strategies to counteract them in DM2 patients.

### Limitations

A key limitation of our study is that our *Drosophila* models rely on overexpression of polyLPAC and polyQAGR peptides, which may induce cellular stress responses or phenotypes that do not fully reflect those in DM2 patient cells. Nevertheless, complementary observations in DM2 patient-derived myoblasts suggest that similar stress-related mechanisms may also occur in a more physiological context, supporting the relevance of our models while acknowledging their limitations. However, the myoblast experiments were performed using cells derived from a single patient with DM2 and a single control individual. Therefore, while these observations are indicative of the conservation of the pathological mechanisms in patient-derived cell lines, a larger study including a greater number of patients with DM2 and matched controls will be required to draw more general and definitive conclusions. The presented data should be interpreted as indicative of inter-species conservation rather than representative of inter-individual variability.

## MATERIALS AND METHODS

### *Drosophila* strains and rearing conditions

*Drosophila* stocks were maintained on standard fly food [25 g/l corn flour, 5 g/l lyophilized agar, 50 g/l sugar, 50 g/l fresh yeast, 2.5 ml/l Tegosept (10% in ethanol) and 2.5 ml/l propionic acid] at 25°C in a 12-h light/dark cycle. All experiments were performed in the same standard conditions, at the temperature reported in figure legends.

Protein-only alternative codon constructs were synthesized by GeneArt (Life Technologies). The plasmids for inducible expression of UAS*-*(*LPAC*)_75_*-3HA* or UAS-(*QAGR*)_75_*-3HA* were generated by cloning the 3HA epitope CDS fused in-frame with the (CCAGCTTGTTTG)_75_ for polyLPAC or (CAGGCTGGACGT)_75_ for polyQAGR into the UAS-attB vector (GeneArt, Life Technologies). The UAS*-(LPAC)_75_-3HA* or UAS*-(QAGR)_75_-3HA* plasmids were injected in *y^1^ w^67c23^; P{CaryP}attP2* embryos (Bloomington *Drosophila* Stock Center, stock #24749); germline transformation was performed by Bestgene Inc. (Chino Hills, CA, USA) using standard procedures. All the driver lines used have been previously described and available from the Bloomington *Drosophila* Stock Center (see Table S1).

### *Drosophila* larval locomotion analyses

Larval locomotor activity was measured by counting the number of peristaltic contractions of third-instar larvae performed within 1 min on the surface of a 1% agarose gel in a Petri dish; measurements were repeated five times for each larva, with at least ten larvae per genotype in each experiment (Coni et al., 2021).

### Survival curves

Control and experimental flies were collected within 24 h of eclosion, sorted by sex under carbon dioxide anaesthesia and maintained in a climate chamber on a standard fly food at constant temperature (25°C). Flies were kept as ten individuals per vial in triplicate. Dead flies were recorded daily, and fresh medium vials were provided twice a week. The Kaplan–Meier method (GraphPad Prism 6) was used for the survival analysis, by constructing a survival curve that tracks the survival rate over time.

### Immunoblotting

#### Drosophila

Protein extracts were derived from third-instar larvae lysed in sample buffer, fractionated by SDS-PAGE and transferred to nitrocellulose membrane. Nitrocellulose membranes were incubated overnight at 4°C with primary antibodies and for 2 h at room temperature with secondary antibodies. Primary and secondary antibodies were diluted in 5% milk in PBS-Tween 0.1% (GE Health Care). Dilutions are reported in Table S1.

#### Human cells

Myoblast cells derived from patients with DM2 and unaffected controls were lysed in denaturing buffer SDS-urea [50 mM Tris HCl, pH 7.8, 2% sodium dodecyl sulphate (SDS), 10% glycerol, 10 mM Na_4_P_2_O_7_, 100 mM NaF, 6 M urea, 10 mM EDTA]. Protein extracts were then sonicated, quantified, and resolved by SDS-PAGE before transfer to a nitrocellulose membrane (NBA085C001EA, Perkin Elmer). Primary and secondary antibodies were diluted in 5% milk in PBS-Tween 0.1% (GE Health Care). Detection was performed using WesternBright ECL (K-12045-D50, Advansta). Signals were detected and acquired using the Gel Doc II scanning system (Bio-Rad). Densitometric analysis of bands was performed using the ImageJ software 1.50i. Primary antisera specifics and dilutions used are reported in Table S1.

### HeLa cell transfection

HeLa cells were grown in Dulbecco's Minimal Essential Medium (DMEM; BioWhittaker) supplemented with 10% fetal bovine serum (FCS; Gibco), 200 mM L-glutamine, penicillin (100 mg/ml) and streptomycin (100 mg/ml) and maintained at 37°C in 5% CO_2_. Cells were transfected using Lipofectamine 2000 (Invitrogen-Thermo Fisher Scientific) according to the manufacturer's protocol.

### Immunofluorescence

#### HeLa cells

For immunofluorescence experiments, cells were washed in 1× PBS and then fixed for 20 min in 4% paraformaldehyde and 4% sucrose, dissolved in 60 mM PBS pH 7.4. After permeabilization in 0.25% Triton X-100 in PBS for 10 min, cells were incubated for 1 h in blocking solution [1× PBS, 0.1% Tween 20, 3% bovine serum albumin (BSA)] and then incubated for 1 h with the indicated primary antibodies in blocking solution in the humidified chamber at room temperature (RT). After three washes in 0.1% Tween 20 in PBS, coverslips were incubated with the secondary antibody for 1 h at RT, washed three times in 0.1% Tween 20 in PBS and mounted with DAPI/VectaShield (Vector Laboratories) on microscope slides. For oxidative stress, cells were exposed to 0.5-1 mM sodium arsenite in complete medium for 30 min at 37°C. Cells were observed using a ZEISS Axioplan microscope equipped with a CCD camera (Photometrics). Images were acquired using the ImageJ and processed with Adobe Photoshop.

#### Larval brains

Third-stage larval brains were dissected in physiological solution, fixed in 3.7% formaldehyde in PBS for 20 min, briefly dipped for 30 s in 45% acetic acid (in ddH_2_O), and then transferred for 2 min onto a coverslip with a drop of 60% acetic acid (in ddH_2_O). Obtained preparations were squashed and flash-frozen in liquid nitrogen. The coverslip was removed, and the slides were immersed in ice-cold absolute ethanol for 10 min and washed twice for 10 min each wash in PBT (0.1% Triton X-100 in 1× PBS). Fixed preparations were incubated overnight with the indicated primary antibodies in PBS in a humid chamber at 4°C. The following day, the slides were washed twice in PBS for 5 min and then incubated for 1 h at RT with secondary antibodies in PBS. After two additional washes in PBS for 5 min, the slides were mounted with DAPI/VectaShield and stored at −20°C. Cytological analysis was performed using a ZEISS Axioplan microscope equipped with a CCD camera (Photometrics). Images were acquired using ImageJ and processed with Adobe Photoshop.

#### Larval muscle

Larvae were dissected in ice-cold Ca^2+^-free HL3 saline and fixed in 4% formaldehyde for 10 min and washed in PBS containing 0.05% Triton X-100 (PBST) for 30 min. Fillets were incubated overnight at 4°C with specific primary antibodies (Table S1). After washing, larval fillets were stained with phalloidin–TRITC (1:300, diluted in PBST; Sigma-Aldrich) for 40 min at RT and subsequently washed for three times, 20 min each wash, with PBST. Larvae were mounted in DAPI/VectaShield. Confocal microscopy was performed with a Leica SP8 confocal microscope. Confocal imaging of larval fillets was performed using a *z*-step of 0.5 μm. The following objective was used: 63×1.4 NA oil immersion for confocal imaging. All confocal images were acquired using the LCS AF software (Leica). Images from fixed samples were taken from third instar larval fillets (segment A2, muscle 6/7).

#### Larval fat bodies

Larvae were placed in ice-cold 1× PBS, dissected in 4% formaldehyde, and fixed for 30 min. After fixation, open larvae were washed in PBS containing 0.01% Triton X-100 twice, 10 min, each wash, and blocked for 1 h in blocking solution (BS) (3% BSA, 1% normal goat serum in PBT). Then open larvae were incubated overnight at 4°C with anti-HA rabbit (1:250 in BS; Abcam) and anti-Fibrillarin rabbit (1:500 in BS; Gene Tex). The day after, open larvae where washed two times, 10 min, each wash, in PBT and incubated for 2 h at RT with FITC-conjugated goat anti-rabbit (1:50 in PBT; Jackson ImmunoResearch), Alexa Fluor 555-conjugated donkey anti-mouse (1:50 in PBT; Invitrogen) and DAPI (1:300 in PBT). Antibody details are in Table S1. Open larvae were then rinsed in PBT, and the fat bodies isolated, placed on poly-L-lysine slides, and mounted in anti-fade mounting medium. Fat body preparations were analysed on a fluorescence microscope (ZEISS Apotome) and images acquisition performed using Zen Pro software (ZEISS).

#### DM2-derived cells

Primary myoblasts were provided by Vincent Mouly (Institut de Myologie, France) and were derived from the quadriceps muscle of a 53-year-old control female donor and a 53-year-old female patient with DM2 (carrying 4000 CCTG repeats in the muscle; Arandel et al., 2017). Myoblast cells were seeded at a density of 1×10^4^ cells/cm² in CultureSlides chambers (Falcon, 354118, Corning Inc.). Cells were fixed with 4% paraformaldehyde and permeabilized using 0.5% Triton X-100. Following a 30-min blocking step in 3% BSA, cells were incubated overnight at 4°C with the primary antibody. The following day, cells were incubated with the appropriate fluorophore-conjugated secondary antibody and counterstained with Hoechst (1:300; 33342, Invitrogen). Confocal images were acquired at 63× magnification using a LSM 980 confocal laser-scanning microscope equipped with Airyscan 2 (ZEISS).

### RNA extraction and quantitative PCR

Total mRNA was isolated from *Drosophila* larval fat bodies or carcasses using Trizol (15596026, Thermo Fisher Scientific) according to the manufacturer's instructions. RNA was reverse-transcribed (1 mg each experimental point) using the SensiFAST cDNA Synthesis Kit (BIO-65053, Bioline) and qPCR was performed as described (18) using SensiFast Sybr Lo-Rox Mix (BIO- 94020, Bioline). The run was performed using an Applied Biosystems Quant Studio 3 Real-Time PCR System 36 instrument (Marzullo et al., 2023b). Primer sequences are reported in Table S1.

### Image analysis, representation, and statistical analyses

Images of fly eyes were taken with a ZEISS Semi 508, 50X stereomicroscope equipped with Axiocam camera 212 using ZEISS ZEN software (blue edition). Eye area was measured with the open-source software NIH ImageJ Fiji.

Images of *Drosophila* larval fat bodies were analysed using Fiji software. The number of cells with fragmented nucleoli was quantified and expressed as a proportion of the total number of cells per field. Fragmentation was defined based on nucleolar morphology and shape, as identified in the fibrillarin channel. Nucleolar size was measured by outlining individual nucleoli and calculating the corresponding area in Fiji.

To quantify the number of puncta in each image, an arbitrary but fixed region of interest (ROI) of identical size was selected for all images. The analysis was performed three times per image, and the number and size of puncta within each ROI were quantified using the ‘Analyse Particles’ function in Fiji. Cytoplasmic and nuclear signals were quantified using Fiji. Nuclear ROIs were defined based on DAPI staining, and whole-cell ROIs were delineated using LPAC-HA or QAGR-HA signal. Cytoplasmic ROIs were obtained by subtracting the nuclear ROI from the whole-cell ROI. Within each compartment, HA signal intensity and the number of aggregates were quantified using standard Fiji functions (‘Measure’ and ‘Find Maxima’) with identical settings across all samples. Colocalization analysis was performed in Fiji using the JACoP plugin to calculate Manders correlation coefficient from single ROIs of the original images.

The Shapiro–Wilk Test was used to assess the normal distribution of every group of different genotypes. Statistical differences for multiple comparisons were analysed with the Kruskal–Wallis for non-parametric values or with one-way ANOVA for parametric values. The Dunn's or the Tukey's test was performed, respectively, as post-hoc test to determine the significance between every single group. The Mann–Whitney *U-*test or an unpaired one-tailed *t*-test were used for two-group comparison of non-parametric or parametric values, respectively. Statistical significance was defined as *P<0.05, **P<0.01, ***P<0.001, ****P<0.0001 and ‘ns’ for not significant.

### Use of AI

ChatGPT (OpenAI) was used solely for minor English language editing and did not contribute to the development of the scientific content, data analysis, code or figure generation. The authors subsequently reviewed and edited the content as necessary and take full responsibility for the publication's final content.

## Supplementary Material

10.1242/dmm.052729_sup1 Supplementary information
