# Supplementary material for: Distinct cellular effects of myotonic dystrophy type 2 repeat-associated non-AUG tetrapeptides
Source: Dis Model Mech. 2026 May 18;19(5):dmm052729. doi: 10.1242/dmm.052729 (PMC13225714; doi:10.1242/dmm.052729)
Supplement: Supplementary information [file dmm-19-052729-s1.pdf]

Table S1. Key Resources

Reagent type (species) or resource	Designation	Source or reference	Identifiers	Additional information
Genetic reagent (*D. melanogaster*)	*elav-GAL4*	Bloomington	77894	*y[1] w[*] P{w[+mC]= elav(FRT.ewg)GAL4.eFeG}1*
Genetic reagent (*D. melanogaster*)	*D42-GAL4*	Bloomington	8816	*w[*];P{w[+mW.hs]= GawB}D42*
Genetic reagent (*D. melanogaster*)	*Mef2-GAL4*	Bloomington	26882	*w[*]; Kr[If-1]/CyO, P{w [+mC]= GAL4Mef2.R}2,P{w [+mC]=UASmCD8.mRFP}*
Genetic reagent (*D. melanogaster*)	*GMR-GAL4*	Bloomington	9146	*w[1118];P{GMR-GAL4. w[-]}2/CyO*
Genetic reagent (*D. melanogaster*)	*eyeless-GAL4*	Bloomington	5576	*wP{ry[+t7.2]=ey-FLP.N}5;ry[506]*
Genetic reagent (*D. melanogaster*)	*ppl-GAL4*	Bloomington	58768	w[*]; P{w[+mC]= pplGAL4.P}2
Genetic reagent (*D. melanogaster*)	*UAS-QAGR-3HA*	This study	-	injection stock #BL 24749
Genetic reagent (*D. melanogaster*)	*UAS-LPAC-3HA*	This study	-	injection stock #BL 24749
Genetic reagent (*D. melanogaster*)	*UAS-Ref(2)P^{RNAi}*	Bloomington	36111	y[1] sc[*] v[1] sev[21]; P{y[+t7.7]v[+t1.8]=TRiP.HMS00551}attP2
Genetic reagent (*D. melanogaster*)	*UAS-Ref(2)P^{OE}*	de Castro et al., 2013	-	*UAS-Ref(2)P-HA*
Genetic reagent (*D. melanogaster*)	*UAS-Atg5^{RNAi}*	Bloomington	27551	y[1] v[1]; P{y[+t7.7] v [+t1.8]=TRiP.JF02703}attP2
Genetic reagent (*D. melanogaster*)	*UAS-Atg7^{RNAi}*	Bloomington	27707	y[1] v[1]; P{y[+t7.7] v [+t1.8]=TRiP.JF02787}attP2
Genetic reagent (*D. melanogaster*)	*UAS Atg8^{RNAi}*	Bloomington	58309	*y[1] v[1]; P{y[+t7.7] v [+t.8]=TRiP.HMJ22416}attP40*
Genetic reagent (*D. melanogaster*)	*UAS Atg8^{OE}*	Bloomington	37750	*y[1] w[1118]; P{w[+mC]= UASp-mCherry-Atg8a}2; Dr[1]/ TM3,Ser[1]*
Genetic reagent (*D. melanogaster*)	*UAS Rox8^{RNAi}*	Bloomington	28649	Y1 v1;*P{TRiP.JF02870}attP*
Genetic reagent (*D. melanogaster*)	*UAS Rox8^{OE}*	Bloomington	92835	*w[*]; P{UAS-Rox8.3xFlag-HA}attP40/CyO*
antibody	anti-vibrator (rabbit)	Coni et al., 2021	-	WB 1:3000
antibody	anti-H3 (rabbit)	Abcam	Ab18521	WB 1:5000
antibody	anti-HA (rabbit)	Cell signalling technologies	3724	WB 1:500; IF 1:300
antibody	anti-Fibrillarin (mouse)	Abcam	ab5821	WB 1:300
antibody	Actin-Stain 555 Phalloidin	Cytoskeleton	PHDH1-A	IF 1:500

Disease Models & Mechanisms • Supplementary information

antibody	Anti-GABARAP +GABARAPL1+ L2 [EPR4805] (rabbit)	Abcam	ab109364	IF 1:500
antibody	Alexa Fluor 555 Anti-GABARAP +GABARAPL1+ L2 [EPR4805] (rabbit)	Abcam	ab319959	IF 1:500
antibody	anti-Ref(2)P (rabbit)	Abcam	ab178440	WB 1:500
antibody	anti-TIAR (mouse)	Santa Cruz Biotechnology	sc-398372	IF 1:150
antibody	anti-polyLPAC	Sigma-Aldrich	ABN2258	WB 1:1000
antibody	anti-polyQAGR	Sigma-Aldrich	ABN2271	WB 1:1000
antibody	anti-rabbit HRP	Cytiva	RPN4301	WB 1:5000
antibody	anti-mouse HRP	Sigma-Aldrich	NA93IV	WB 1:5000
antibody	anti- rabbit FITC	Jackson ImmunoResearch	111-096-003	IF 1:50
antibody	anti-mouse AlexaFluor 594	Invitrogen	A11032	IF 1:50
antibody	Alexa Fluor 555-conjugated donkey anti-mouse	Invitrogen	A21202	IF 1:50
Dye	Hoechst	Invitrogen	33342	IF 1:300
primer	*FW_pre-rRNA*	Biofab research	-	TTTGAACACGGACT TGGCT
primer	*RV_pre-rRNA*	Biofab research	-	GCCTGCCACCAAAAA TTAACG
primer	*FW_rRNA_28S*	Biofab research	-	ATTAACACAATCCCG GGGCG
primer	*RV_rRNA_28S*	Biofab research	-	GGTACGTTCCAGTTA GAGGCA
primer	*FW_rRNA_18S*	Biofab research	-	GGTGCTGAAGCTTAT GTAGCCT
primer	*RV_rRNA_18S*	Biofab research	-	GACAAACCAACAGGT ACGGC
primer	*FW_rRNA_5.8S*	Biofab research	-	CTAAGCGGTGGATCA CTCGG
primer	*RV_rRNA_5.8S*	Biofab research	-	TGTCCTGCAGTTCACA CGAT
primer	*FW_rRNA_*Atg5	Biofab research	-	ATTAAGCCGGAGCCTTT CTATC
primer	*RV_rRNA_*Atg5	Biofab research	-	ATCGCCATACGGTTCCA TTAG
primer	*FW_rRNA_*Atg7	Biofab research	-	GGAATGCTGTGCAACTA CGA

Disease Models & Mechanisms • Supplementary information

primer	*RV_rRNA*_Atg7	Biofab research	-	GGAGATTCCCGTCAA ATCCT
primer	*FW_rRNA*_Atg8	Biofab research	-	AGGATGCCCTCTTCTT CTTTG
primer	*RV_rRNA*_Atg8	Biofab research	-	GCTAACTCGCCGTCC ATATT
primer	*FW_rRNA_ Ref(2)P*	Biofab research	-	CCTTCTGTCGACGATC CGAG
primer	*RV_rRNA_ Ref(2)P*	Biofab research	-	GGCATGCGTAGCATC AAGTG

Disease Models & Mechanisms • Supplementary information
